# Influence of Surface Roughness on Biodegradability and Cytocompatibility of High-Purity Magnesium

**DOI:** 10.3390/ma15113991

**Published:** 2022-06-03

**Authors:** Jiahao Chen, Jingtao Dai, Junyu Qian, Weirong Li, Ronghui Li, Dong Pang, Guojiang Wan, Ping Li, Shulan Xu

**Affiliations:** 1 Department of Oral Implantology, Stomatological Hospital, Southern Medical University, Guangzhou 510280, China; graholl@smu.edu.cn (J.C.); jingtaodai_88@smu.edu.cn (J.D.); 2 Key Laboratory of Advanced Technologies of Materials, Ministry of Education, School of Materials Science and Engineering, Southwest Jiaotong University, Chengdu 610031, China; junyu.qian@my.swjtu.edu.cn (J.Q.); guojiang.wan@home.swjtu.edu.cn (G.W.); 3 Medical Device Division, Dongguan Eontec Co., Ltd., Dongguan 523662, China; liwr@e-ande.com (W.L.); lironghui@e-ande.com (R.L.); pangd@e-ande.com (D.P.)

**Keywords:** biodegradable metals, magnesium, cytotoxicity, biodegradability, surface roughness

## Abstract

High-purity magnesium (Mg) is a promising biodegradable metal for oral and maxillofacial implants. Appropriate surface roughness plays a critical role in the degradation behavior and the related cellular processes of biodegradable Mg-based metals. Nevertheless, the most optimized surface roughness has been questionable, especially for Mg-based oral and maxillofacial implants. Three representative scales of surface roughness were investigated in this study, including smooth (Sa < 0.5 µm), moderately rough (Sa between 1.0–2.0 µm), and rough (Sa > 2.0 µm). The results indicated that the degradation rate of the Mg specimen in the cell culture medium was significantly accelerated with increased surface roughness. Furthermore, an extract test revealed that Mg with different roughness did not induce an evident cytotoxic effect. Nonetheless, the smooth Mg surface had an adversely affected cell attachment. Therefore, the high-purity Mg with a moderately rough surface exhibited the most optimized balance between biodegradability and overall cytocompatibility.

## 1. Introduction

Bone defect occurs in the oral and maxillofacial area for various reasons, including trauma, tumor, and congenital anomaly. Due to its complex anatomical structure, clinicians find it difficult to regenerate the bone tissue and restore function and aesthetics [1]. Biocompatible materials such as titanium [2,3], bioactive glass [4], and synthetic polymer [5] can be applied for reconstructing the craniomaxillofacial tissue; however, the poor mechanical properties of the bioactive glass limit the loading application [6]. The synthetic polymer has a slower degradation rate compared to the healing process of tissue formation. More importantly, the synthetic polymer exhibits a relatively weak cellular response due to its hydrophobicity and could release acidic byproducts [7]. Titanium-based (Ti) implants possess excellent mechanical properties; however, based on oral and maxillofacial applications, the stiffness of the Ti-based implant (i.e., Ti mesh) can lead to mechanical stimulation of the mucosal flap, causing an increased exposure risk; thereby, the additional surgery to remove the implant is inevitable [8].

Biodegradable metals (BMs) refer to a metal or alloy intended to degrade in vivo [9]. Magnesium (Mg)-based BMs have been considered promising maxillofacial implant materials due to their excellent biocompatibility, superior mechanical strength, appropriate biodegradability, and sufficient bioactivity [10,11]. Notably, high-purity Mg possesses superior biocompatibility, and the released Mg ions can enhance the viability of human osteoblasts, alkaline phosphate activity, and osteocalcin levels [12]. In addition, the degradation products of Mg and its alloys are phagocytosed by the macrophages and safely excreted through urine [13,14]. Previous in vivo studies demonstrated that high-purity Mg implants could effectively reconstruct bone tissues and blood supplies [15,16]. To date, the pre-clinical application of Mg-based implants primarily includes bone screws, plates, and screw systems [17,18,19]; however, Mg and its alloys generate hydrogen gas at the early implantation stage, forming gas cavities [10]. Numerous studies have focused on improving alloying [20,21], coating [22,23], and surface modification techniques [24] to regulate degradation rate and the related biological response.

Surface roughness is a critical parameter in predicting the corrosion behavior in metals [24,25,26]. Scratches on the surface of metals and corrosion pits could cause stress rise, which allows a decrease in resistance to fracture, leading to stress corrosion cracking or hydrogen embrittlement, causing premature failure of biodegradable implants during service [27,28,29]; however, there have been inconsistent reports regarding the impact of roughness on the degradation behavior of Mg and its alloys. On the one hand, for the Mg-based alloy, the degradation rate of the polished surface was more significant than the semi-polished surface of Mg-based alloys due to more general corrosion occurring [30]. Initially, pitting corrosion appeared on the rough surface during the early stage. After 12 h immersion, the rough surface presented general and localized corrosion, while localized corrosion began on the smooth surface; however, no significant effect of surface roughness was detected under exposure within the simulated body fluid for 12 h [31]. On the other hand, Nguyen et al. reported that the degradation rate of pure Mg was accelerated with increasing surface roughness, without any pitting corrosion [32]. Similarly, a recent critical review about the effect of surface roughness on the degradation derived that mechanical surface treatment on Mg and its alloys existed a trend of increasing degradation rate with greater surface roughness [33]; however, to our knowledge, very few reports exist on the degradation behavior of high-purity Mg with different surface roughness, especially on oral and maxillofacial applications.

Extracellular matrix topography is one of the essential physical cues for determining cell adhesion and differentiation [34]. The surface topography of an implant is the process of integrating and converting physical signals in microecology into intracellular biochemical signals recognized by cells [35]. Previous studies investigated the effect of surface roughness topography on modulating cell behaviors. For instance, the highest surface roughness of titanium depicted a high capacity for cell proliferation and cell attachment [36]; however, Khang et al. reported that a rougher titanium surface simulated cell proliferation [37]. The degradation behavior can affect the release of degradation products and generate cytotoxic effects on cell activity [25]. Adding alloying elements to pure magnesium causes micro-galvanic corrosion, leading to an increasing degradation rate of Mg alloys, which finally increases pH value and the release of metallic ions exceeding the cellular tolerance [38]. Naked Mg or improper surface modification may lead to excessive degradation, increasing Mg ion and hydroxide ion release, causing cell apoptosis. It was reported that the sample extract with Mg ion < 3.85 mM had minor inhibition in endothelial cells [24] and extracts with Mg ion concentration of 7.2 mM affected the cellular activity of the human umbilical vein endothelial cells [39]; however, the role of high-purity magnesium with different surface roughness in regulating cytocompatibility has not been elucidated.

This study is, to the best of our knowledge, the first report on the effect of different surface roughness on the in vitro degradation behavior and cytotoxicity of biodegradable high-purity Mg. The high-purity Mg was selected as an investigated material due to its excellent biosafety and bioactivity, superior corrosion resistance, and sufficient mechanical, as previously published in detail [15,40,41]. Based on the surface scales of the craniomaxillofacial implants, three representative scales of surface roughness were evaluated, including smooth (Sa < 0.5 µm), moderately rough (Sa between 1.0–2.0 µm), and rough (Sa > 2.0 µm), respectively [42]. In addition, the in vitro degradation behavior was assessed using an immersion test. Moreover, the cytotoxicity evaluation was investigated using the extract test and direct contact test. The first null hypothesis indicated that representative different surface roughness exerted no influence on the in vitro degradation behavior of pure Mg. The second null hypothesis indicated that different surface roughness of pure Mg would not affect the cytocompatibility.

## 2. Materials and Methods

### 2.1. Specimen Preparation

High-purity magnesium (purity 99.99%, 10 mm in diameter, 2 mm in thickness) was fabricated as described previously (Eontec. Co., Ltd., Dongguan, China), which compose of 99.99 wt% Mg, Al 0.002 wt%, Si < 0.001 wt%, Ca < 0.001 wt%, Ti < 0.0001 wt%; Mn < 0.002 wt%; Fe < 0.001 wt%; Ni < 0.0001 wt%, Cu < 0.0002 wt%, Zn < 0.0028 wt%, and Pb < 0.0008 wt%, as previously reported [15]. Mechanical grinding was one of the methods to develop the surface roughness gradient attributes of biodegradable metals. The uniform texture of surface attributes was obtained by auto mechanical grinding [43,44]. The entire surface of the specimens was grounded with silicon carbide (SiC) abrasive paper (EXTEC, Enfield, CT, USA) using a mechanical grinding machine (EXAKT, Norderstedt, Germany). Specifically, the specimens were ground with the SiC abrasive paper up to P180, P400, and P4000, respectively, based on the different scales such as smooth, moderately rough, and rough. Next, the specimens were cleaned ultrasonically using absolute ethanol for 15 min and dried on a sterile workbench. Each specimen surface was, respectively, sterilized with ultraviolet for at least 30 min before the tests.

### 2.2. Surface Characterization

Prior to the surface observation, sample surfaces were sputtered-coated with a 20 nm thick gold-palladium. The surface morphology was characterized by using a scanning electron microscope with an energy-dispersive X-ray spectroscopy instrument (SEM-EDS, MIRA4 LMH, TESCAN, Brno, Czech Republic) at an acceleration voltage of 15 kV. The surface roughness was determined by an optical profiler (Bruker Countor GT K 3D, Billerica, MA, USA). Four specimens per group were measured with vertical scanning interferometry with a 1× magnification lens, a field of view of 0.4 × 0.4 mm, and a scan speed of ×1. According to the manufacturer’s instructions, the ‘VXI’ mode was used to reduce the noise level in the flat area. Moreover, the tested areas were 3D reconstructed for visualizing the surface topographies. According to ISO 25178: 2012 [45], the height, spatial, and hybrid surface texture parameters were chosen to describe the characteristics of the implant topographies. Based on the surface scales of the craniomaxillofacial implants, the arithmetical mean height (Sa), root mean square height (Sq), texture aspect ratio (Str), and developed interfacial area ratio (Sdr) were selected, as previously reported [46]. All parameters were determined and analyzed using the Vision 64 software (Bruker, Billerica, MA, USA).

### 2.3. Immersion Test

The in vitro degradation behavior was investigated using an immersion test with different simulated body fluids. A cell culture medium and artificial saliva were chosen to be the electrolytes for the tests to mimic the submucosa and intraoral environments of implantation sites. All specimens were immersed under cell culture conditions (5% CO_2_, 95% humidity, 37 °C) for 30 days. According to ISO 10993-12: 2012 [47], the ratio of solution volume to sample surface area was 1 mL: 1.25 cm^2^. According to the information from the manufacturer (Leagene Biotechnology, Beijing, China), the pH value of artificial saliva was adjusted to ~5.5 by hydrochloric acid, and the components are shown in Table 1. Cell culture medium was prepared using the Dulbecco’s modified Eagle medium (DMEM, Gibco, Grand Island, NY, USA) with 10% fetal bovine serum (FBS, ExCell Bio, Shanghai, China) [48].

Eight parallel specimens were taken with the cell culture medium and artificial saliva for each group. The immersion solution was refreshed every two days to perform a semi-static degradation process. Meanwhile, the pH value was measured at each time point. After 30 days of immersion, samples were taken out from the immersion solution and gently rinsed with distilled water. Next, the degradation products were removed by a chromic acid solution (180 g CrO_3_ dissolved in 1000 mL distilled water, Macklin, Shanghai, China) for 20 min at room temperature, as previously reported [49]. Afterward, the samples were rinsed with distilled water and dried with an air stream. The samples (n = 4) per group were used to determine the degradation rate, according to ASTM G1-03: 2017 [50]. The degradation rate was calculated and expressed as μm/year as per Equation (1):

(1) $$\mathrm{Degradation}\;\mathrm{rate}\;(\mu m/\mathrm{year})=8.74\;\times \;10^{7}\frac{\Delta W}{A\;\times \;t\;\times \;\rho }$$

where ΔW (g) is the mass loss before and after removal of the corrosion products; *A* is the exposure area of sample (cm^2^); t is the immersion time (h); ρ is the density of material (1.74 g/cm^3^). Additionally, sample morphology before and after removal of degradation products was characterized by SEM-EDS.

**Table 1:** Main composition of artificial saliva according to the manufacturer.

Composition	Concentration
Sodium chloride	0.40 g/L
Potassium chloride	0.40 g/L
Calcium chloride dehydrate	0.79 g/L
Sodium dihydrogen phosphate dihydrate	0.78 g/L
Urea	1.00 g/L
Sodium sulfide	0.05 g/L

### 2.4. Cytotoxicity Test

#### 2.4.1. Cell Culture

Mouse fibroblasts (L929, Procell Life Science and Technology Co., Ltd., Wuhan, China), mouse preosteoblast cells (MC3T3-E1, Procell Life Science and Technology Co., Ltd., Wuhan, China), and mouse macrophages (RAW264.7, Procell Life Science and Technology Co., Ltd., Wuhan, China) were used to investigate the cytotoxicity of specimens. In a standard cell incubator (5% CO_2_, 95% humidity, 37 °C), three cell lines were cultured in Dulbecco’s modified Eagle medium (DMEM, Gibco, Grand Island, NY, USA) with 10% fetal bovine serum (FBS, Gibco, Grand Island, NY, USA) and penicillin and ptreptomycin (100 U/mL) (PS, Gibco, Grand Island, NY, USA). The complete cell medium was refreshed every two days. Cell passage was carried out when cells reached about 80% confluency.

#### 2.4.2. Extract Test

An extract test was performed based on ISO 10993-5: 2009 [51] and ISO 10993-12: 2012 [47] guidelines. Specifically, the surface area ratio and sample extract were set to 1.25 cm^2^/mL for 72 h under cell culture conditions. In addition, the titanium-based alloy was set as the negative control while the pure copper became the positive control. Meanwhile, a pH meter (SX-620, Sanxin, Shanghai, China) was used to measure the pH value of specimens extracts. The concentration of Mg ion was measured by atomic absorption spectrophotometry (TAS-990F, Persee Inc., Beijing, China).

The live/dead cell fluorescence staining was undergone to qualitatively analyze cytotoxic effects using a live/dead viability/cytotoxicity assay kit (KGAF001, KeyGEN BioTECH, Nanjing, China). Three types of cell lines were cultured with a cell culture medium in a 12-well plate at a density of 3 × 10^4^ cells/mL overnight. Afterward, the medium was exchanged with sample extracts. Then, the extracts were removed after incubation for 24 h, and gently rinsed the cells with phosphate-buffered saline (PBS, Gibco, Grand Island, NY, USA). Subsequently, 2 mL of staining reagent containing 2 μM calcein acetoxymethyl (Calcein AM) and 8 μM propidium iodide (PI) were added and cultured for 10 min in the dark at room temperature. Furthermore, cell morphology and viability were observed by downright fluorescence microscopy (DMi8, Leica Microsystems GmbH, Wetzlar, Germany). Three parallel wells per group were set, and at least three fields of each well were randomly selected to shoot.

To further determine the relative cell viability and cell membrane integrity, a cell counting kit-8 assay (CCK-8, Dojindo Laboratories Co., Kumamoto, Japan) and an lactate dehydrogenase (LDH) cytotoxicity assay kit (Beyotime Biotechnology, Jiangsu, China) were performed, respectively. Briefly, three cell lines were individually seeded into a 96-well plate at a density of 3 × 10^4^ cells/cm^2^. After incubation for 24 h, the cell culture medium was interchanged with 100 μL sample extracts. After 24 h, the extract was removed and added 100 μL DMEM without FBS, and 10 μL CCK-8 reactant was added for 2 h. At the time point, a microplate reader (iMark, Bio-rad, Hercules, CA, USA) was used to record the absorbance value at 450 nm. The relative metabolic activity was calculated according to Equation (2).

(2) $$\mathrm{Relative}\;\mathrm{metabolic}\;\mathrm{activity}\;(\%)=\frac{OD_{samples}-OD_{blank}}{OD_{negative}-OD_{blank}}\times 100\%$$

where $OD_{samples}$ means optical density (*OD*) value of samples; $OD_{negative}$ means the *OD* value of the negative control and $OD_{blank}$ means the *OD* value of DMEM alone with CCK-8 reagent.

The LDH cytotoxicity assay kit was utilized as per the manufacturer’s instructions. Briefly, three cell lines were seeded onto the 96-well plates and the cell culture medium was exchanged with sample extracts, as mentioned above. Before 1 h of the testing timepoint, the LDH release reagent was added to the wells of maximum cell enzyme activity, then cultured in the incubator for 1 h. Subsequently, the 96-well plate was centrifuged with a plate centrifuge (PlateSmart, Miulab, Hangzhou, China) for 5 min at 400 g. A total of 120 μL supernatant per well was shifted to a new 96-well plate and incubated with a 60 μL LDH reaction reagent. After incubating in the dark at room temperature for 40 min, the absorbance was determined at 490 nm using a microplate reader (iMark, Bio-rad, Hercules, CA, USA). The relative LDH release was calculated according to Equation (3).

(3) $$\mathrm{Relative}\;\mathrm{LDH}\;\mathrm{release}\;(\%)=\frac{OD_{samples}-OD_{blank}}{OD_{max}-OD_{blank}}\times 100\%$$

where $OD_{samples}$ is OD value of samples; $OD_{max}$ is OD value of the maximum cell enzyme activity and $OD_{blank}$ is the OD value of DMEM alone with LDH reaction reagent.

#### 2.4.3. Direct Contact Test

To mimic the initial protein absorption and the formation of degradation layer, the specimens with different roughness were pre-incubated with the cell culture medium for 24 h, as previously reported [52]. Afterward, the mouse macrophages were seeded on the materials at a density of 3 × 10^4^ cells/cm^2^ into the 12-well plate for 24 h. Three parallel samples per group were utilized for each experiment. The Calcein AM/PI staining was used to determine cell membrane integrity. In addition, the qualitative and quantitative results were evaluated using a fluorescence microscopy and flow cytometry, respectively.

Live/dead cell fluorescence staining was performed to qualitatively analyze cell membrane integrity which is directly in contact with materials. Similarly, as mentioned above, RAW264.7 cells were seeded on pre-treated samples in a 12-well plate for 24 h. Afterward, specimens were stained with 10 mL PBS containing 5 μL of 16 μM Calcein AM and 5 μL of 8 μM PI for 10 min after gently rinsing with PBS. The cell membrane was observed by the confocal fluorescence microscope (DMi8, Leica Microsystems CMS GmbH, Wetzlar, Germany).

The percentage of live cells was analyzed quantitatively using flow cytometry. Briefly, the supernatant of extracts was collected into centrifuge tubes. After washing and trypsinization, the cells in PBS and Trypsin-EDTA (Gibco, Grand Island, NY, USA) were pooled and centrifuged (1000 rpm for 5 min), followed by resuspension in 1 mL PBS. To identify the dead cells, 200 μL cell suspensions were incubated with 1 μL of 1 g/L PI reagent (10 mg PI in 10 mL ddH_2_O, Sigma-Aldrich, Stein-Heim, Germany) at 4 °C in the dark for 1 min before measurement. Subsequently, the cell viability was assessed using the flow cytometry instrument (DxFLEX, Beckman Coulter, Suzhou, China).

### 2.5. Statistical Analysis

All the quantitative data were presented as the mean and standard deviation. To ensure reproducibility, all experiments were independently performed at least three times. Statistical analyses were performed by using GraphPad Prism 9 (GraphPad Software, Inc., San Diego, CA, USA). One-way analysis of variance (ANOVA) was utilized to analyze the surface roughness, degradation test, and cytotoxicity test, followed by Tukey’s comparisons test. Statistical difference was regarded as significant when the *p*-value was less than 0.05.

## 3. Results

### 3.1. Surface Morphology and Roughness

**Figure 1:**
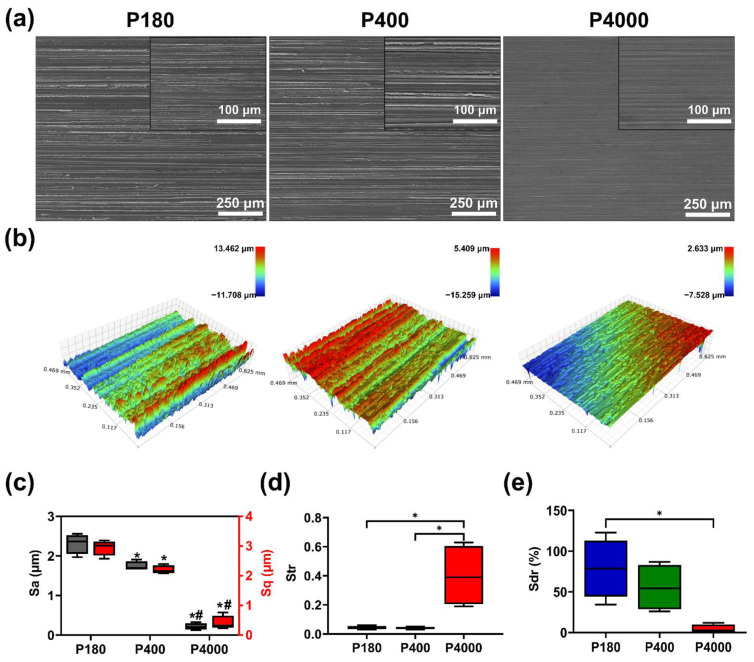
Surface morphology and surface roughness of specimens. (**a**) Representative SEM magnification of different specimens (magnification 100×, scale bar = 250 μm). The inset images corresponded to the magnified surface morphologies (magnification 500×, scale bar = 100 μm). (**b**) Reconstructed three-dimensional surface of specimens with different surface roughness. (**c**) Arithmetical mean height (Sa) and root mean square height (Sq). * and # represent a statistical difference (*p* < 0.05) when compared with the P180 and P400, respectively. (**d**) Texture aspect ratio (Str). * represents a statistical difference between the two groups. (**e**) Developed interfacial area ratio (Sdr). * represents a statistical difference between the two groups.

Surface morphology and three-dimensional reconstruction surface of the samples are presented in Figure 1. The representative SEM image revealed that samples with P180 and P400 exhibited grinding textures in a regular direction (Figure 1a). Moreover, several deep cavities could be observed between the grinding textures, while the sample with P4000 depicted a slight scratch. Furthermore, the interval of the scratches on the surface of P4000 was narrower when compared to the P180 and P400 counterparts. In addition, Figure 1b shows the reconstructed three-dimensional surfaces of the samples with different roughness scales. The surfaces with P180 and P400 exhibited amounts of valleys and protrusions relative to the reference plane. The surface of P180 ranged from −11.7 μm to 13.5 μm, while the P400 ranged from −15.3 μm to 5.4 μm. As a representative image of P4000, the reconstruction surface exhibited that the absolute value of the height of the largest pit within the defined area was approximately 7.5 μm, and the most prominent peak height value was approximately 2.6 μm inside the defined area.

Three-dimension roughness parameters are depicted in Figure 1c–e, including the arithmetical mean height (Sa), root mean square height (Sq), texture aspect ratio (Str), and developed interfacial area ratio (Sdr), respectively. Figure 1c depicts the Sa value and Sq value of the surface of the samples. The Sa and Sq values were similar and gradually decreased. One-way ANOVA confirmed the statistical differences in Sa value and Sq value of roughness gradient treatment: Sa (F (2,9) = 166.80, *p* < 0.0001) and Sq (F (2,9) = 138.90, *p* < 0.0001). Tukey’s multiple comparisons test showed that the group of P4000 had a significant decrease in Sa value and Sq value when compared to the other groups (*p* < 0.0001). When the P180 group was compared with the P400 group, Sa values and Sq value was significantly decreased (*p* < 0.05). As depicted in Figure 1d, one-way ANOVA confirmed the statistical differences in Str value (F (2,9) = 11.27, *p* = 0.0035). Tukey’s multiple comparisons test indicated that the P4000 had a significant increase when compared to the P180 (*p* = 0.0068) and P400 (*p* = 0.0065). As shown in Figure 1e, Tukey’s multiple comparison test indicated significant differences in Sdr value between the P180 group (Sdr = 78.53 ± 36.38%) and the P4000 group (Sdr = 4.37 ± 5.22%).

### 3.2. In Vitro Degradation Behavior

**Figure 2:**
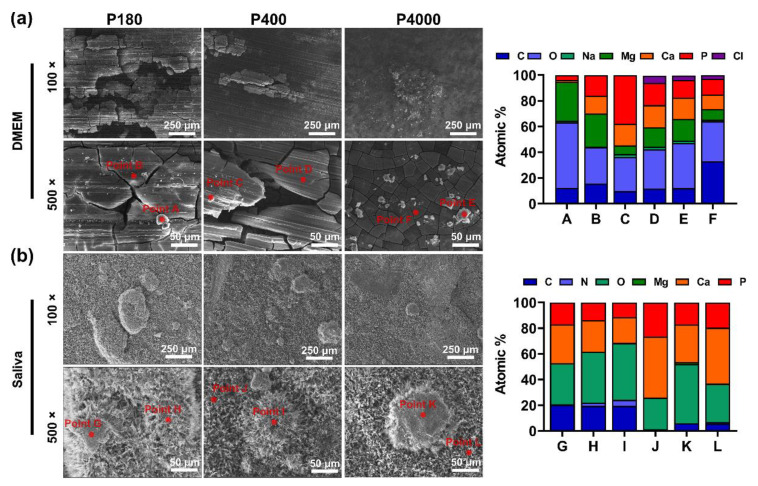
Surface morphology characteristics and chemical composition of samples after immersion test. (**a**) Representative SEM images (scale bar = 250 μm and 50 μm, magnification 100× and 500×, respectively). Surface chemical composition corresponding to the red point A-F of immersing in simulated body fluid. (**b**) Representative SEM images (scale bar = 250 μm and 50 μm, magnification 100× and 500×, respectively). Surface chemical composition corresponding to the red point G-L of immersing in artificial saliva detected by EDS.

To evaluate the long-term degradation of pure Mg samples with different roughness in the simulated body fluids, a long-term immersion test was performed under semi-static conditions. Meanwhile, the degradation kinetics of samples in artificial saliva were also detected. The surface morphology and the chemical composition after different fluids immersion are presented in Figure 2. As illustrated in Figure 2a, after immersion in the simulated body fluids (DMEM + 10% FBS) for 30 days, a multi-sheet-like morphology appears on different roughness surfaces, while grinding textures can be observed on the surface of the P180 and P400 groups. Noteworthily, the area of the lamellar structure presented on the P180 surface is larger than their other two roughness counterparts (P400 and P4000). Meanwhile, the substrate of P180 and P400 groups erupted and presented cavities. The P4000 exhibited similar corrosion products of graininess-like structure, albeit smaller, with an intensive surface distribution. Furthermore, the chemical composition of corrosion products was analyzed using EDS analysis. The EDS results showed that mostly C and O elements could be detected. In contrast, few metal elements were Mg, Ca, and P. As shown in Figure 2b, after immersion in the artificial saliva, a loose pebble-like morphology could be observed, and no textures appeared on the surface. The P180 group surface existed amount of pebble-like corrosion products, while scattered distribution on the P4000 group surface. The C, O, Ca, and P elements were detected by EDS analysis.

**Figure 3:**
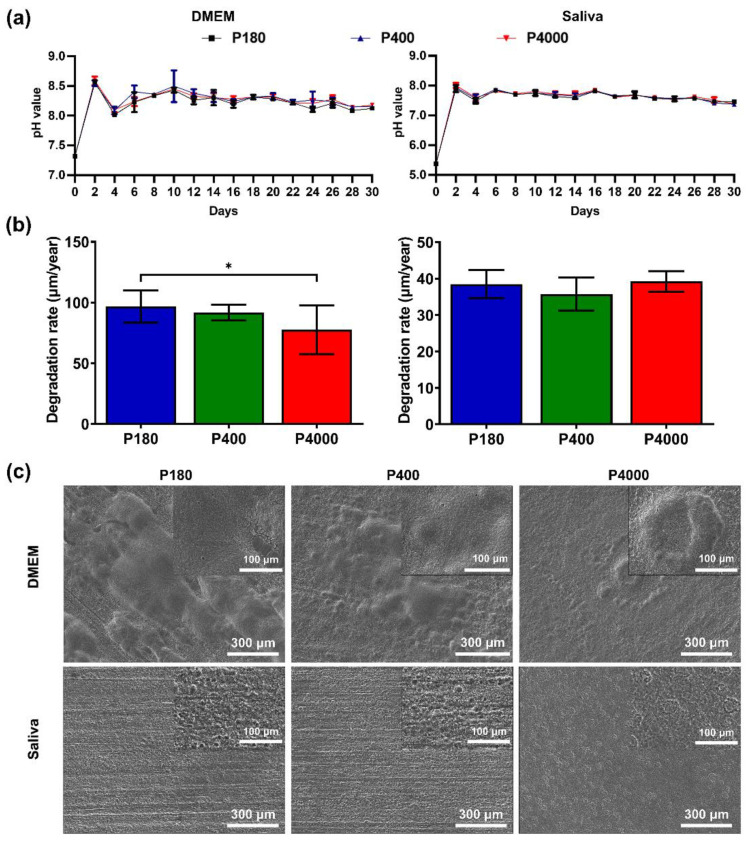
Degradation behavior of pure Mg samples with gradient surface roughness attributes. (**a**) pH value change of simulated body fluid and artificial saliva for 30 days, respectively. (**b**) Degradation rate (μm/year) calculated by weight loss of samples after 30 days of immersion in DMEM and artificial saliva under cell-cultured conditions, where * represents a statistical difference between the two groups. (**c**) Surface morphology of samples after removal of corrosion products. Representative magnification 100× of SEM images and the insets show the magnification 500× of SEM images.

The degradation behavior and morphology of the sample with gradient surface roughness in DMEM and artificial saliva are presented in Figure 3. The samples with different surface roughness appeared to have similar pH value changes in the DMEM and artificial saliva (Figure 3a). Specifically, in the first two-day, pH values dramatically increased from 7.3 to 8.5 and 5.5 to 8.0 of immersion in DMEM and artificial saliva, respectively. Subsequently, pH values tended to be stable, ranging from 8.0 to 8.5 and 7.3 to 8.0. After immersion for 30 days, the degradation rate was obtained by weight loss (Figure 3b). The statistical differences in the degradation rate of samples immersed in DMEM were confirmed by one-way ANOVA (F (2, 33) = 5.69). Tukey’s multiple comparisons test presented that the group of roughest surfaces (P180) had significantly increased degradation rates when compared to the smoothest surface (P4000) (*p* = 0.0073). In addition, a nearly reached significance (*p* = 0.0550) was observed between P400 and P4000 groups. Regarding the samples in artificial saliva, no significant differences in degradation rate were observed based on the results of Tukey’s multiple comparisons.

Figure 3c presents the representative degraded morphology after the removal of the corrosion products. The P180 and P400 samples in DMEM + 10% FBS depicted general corrosion, and several corroded pits could be detected on the surface, while the P4000 group appeared lamellar localized corrosion. The P180 and P400 groups in artificial saliva presented an analogous corrosion morphology after a long-term immersion. The grinding scratch could still be observed after the removal of the corrosion products, and general deep pitting corrosion existed on the sample surface. For the smooth surface of P4000, the area of pitting corrosion was more extensive and shallower compared to the other two different roughness surfaces.

### 3.3. Cytocompatibility Evaluation

**Figure 4:**
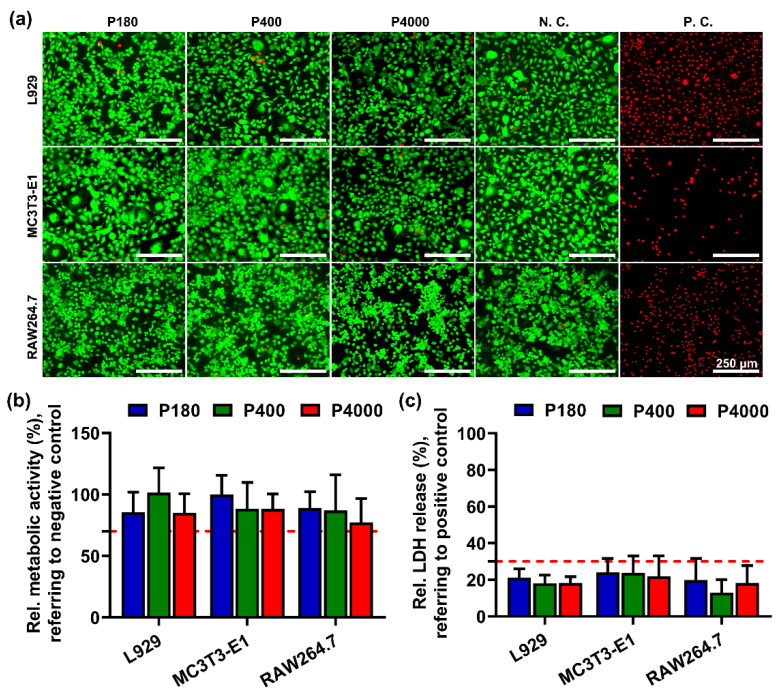
Cytotoxicity evaluation of specimens with different roughness surfaces. (**a**) Representative fluorescent staining images of L929, MC3T3-E1, and RAW264.7 cultured in sample extracts for 24 h (magnification 100×, scale bar = 250 μm). Titanium-based alloy extracts were used as a negative control (N.C.), and pure copper extracts as a positive control (P.C.). The viable cells present green fluorescence while the dead cells show red fluorescence. (**b**) Relative cell metabolic activity of L929, MC3T3-E1, and RAW264.7 after culturing in sample extracts for 24 h detected by CCK-8 assay. Red line indicates 70% of negative control. (**c**) Relative LDH release of three types of cells for 24 h. Maximum cell LDH release was used as a positive control and set to 100%. Dashed line indicates 30% of control, suggesting a line of cytotoxicity effect.

To investigate the cytotoxicity of different samples, three types of cells were cultured in sample extracts. Figure 4a depicts that the cell membrane integrity of L929, MC3T3-E1, and RAW264.7 was exposed to sample extracts. The green fluorescent viable cells were stained using Calcein AM, implying cell membrane integrity. On the contrary, the apoptotic cells presented red fluorescent. Most L929 fibroblasts exposed to pure Mg sampled extracts depicted green fluorescent staining, in line with the negative control. Regarding the other cells exposed to sample extracts, most of them survived (green fluorescent staining) while a few red fluorescent staining (dead cells) was observed. The L929 and MC3T3-E1 cells appeared to have a typical spindle-like morphology, while the RAW264.7 grew contiguously.

To quantitatively determine cell viability, the relative metabolic activity and relative LDH release of different types of cells were measured by CCK-8 assay (Figure 4b) and LDH release assay (Figure 4c), respectively. As shown in Figure 4b, all types of cells exposed to sample extracts above 70% of the negative control implied relatively high metabolic activities. As illustrated in Figure 4c, cells exposed to the sample extracts of different roughness exhibited an appearance below 30% of LDH release relative to the positive control, reflecting no cytotoxic effect of the test extract. Table 2 shows the analysis result of the sample extracts. The Mg ion concentration was detected. Compared to the cell culture medium, Mg ion concentration was significantly elevated among the P180, P400, and P4000 sample extracts. No significant difference was detected in the pH value of the different roughness sample extracts.

**Figure 5:**
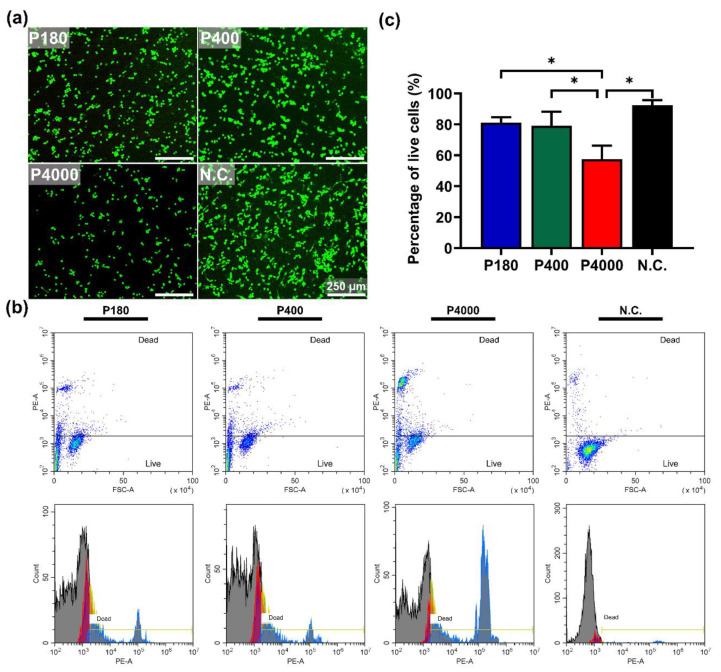
Direct contact test of sample with different roughness. (**a**) Fluorescent staining images of RAW264.7 cultured on sample surfaces (magnification 100×, scale bar = 250 μm). (**b**) The result of flow cytometry, suggesting cell apoptosis of RAW264.7 directly cultured on sample surface, determined by PI staining. (**c**) The quantitative results of cell viability. Titanium-based alloy was set as a negative control (N.C.). * represents a statistical difference between the two groups.

Cells were directly cultured on the textured surface of pure Mg samples having different roughness attributes for 24 h. Figure 5a reveals that the cells cultured on the P180 and P400 samples had benign reactive and suitably distributed morphologies, comparable with the negative control. Regarding the P4000 samples, the green fluorescent staining (live cells) significantly decreased, indicating exist cytotoxic effect. The red fluorescent staining was hardly detected due to the dead cells being removed when rinsing the samples before the fluorescent stain. Figure 5b,c show the cell apoptosis of RAW264.7 directly cultured on the sample surface, determined by PI staining flow cytometry. The percentage of live cells of macrophages cultured on the P4000 sample surface was below 70%. One-way ANOVA was used to confirm the statistical differences in the percentage of live cells cultured on the sample surfaces. Tukey’s multiple comparisons showed significant differences in the percentage of live cells among the cells cultured on the smooth surface of pure Mg samples (57.44 ± 8.773%) and the negative control (92.42 ± 3.266%, *p* < 0.0001). The percentage of live cells directly attached to the pure Mg samples with different surface roughness showed significant differences when comparing P4000 samples (57.44 ± 8.773%) with P180 samples (81.09 ± 3.572%, *p* = 0.0002) and P400 samples (79.16 ± 9.074%, *p* = 0.0006).

## 4. Discussion

Biodegradable Mg and Mg-based alloys have been categorized as a new generation of promising implant materials for craniomaxillofacial applications thanks to their superior biodegradability and biocompatibility. Roughness surface is a critical parameter for optimizing the materials’ properties of Mg-based metals. Nonetheless, the ranges of the most optimized roughness for Mg-based implants remain unclear. The current study investigated the effect of different surface roughness on the in vitro degradation behavior and the cytocompatibility of high-purity Mg. In this study, the surface textures with different roughness range, such as Sa value > 2.0 µm, Sa value between 1.0 µm and 2.0 µm, and Sa value < 0.5 µm, were obtained by automatic grinding machine. Meanwhile, in comparing the surface texture to the definition area, the surface area increased by approximately 80%, 55%, and 4%, correspondingly. Our results indicated that the degradation rates in DMEM were significantly elevated by surface roughness (*p* < 0.01). In addition, a direct contact test revealed that it significantly affected macrophages adhesion (*p* < 0.01); therefore, both hypotheses were rejected.

### 4.1. Biodegradability Influenced by Surface Roughness

The in vitro degradation behavior of pure Mg was investigated through a semi-static immersion test with two different simulated body fluids, namely DMEM with 10% FBS and artificial saliva. Previous studies have reported that DMEM with 10% FBS under cell culture conditions could mimic in vivo physiological implantation environment, especially the interstitial fluid [48,54,55]. In addition, considering the different maxillofacial applications (i.e., guided bone regeneration membrane), the implants could have exposure to the oral environments, indicating switching of the tissue fluid to human saliva; therefore, artificial saliva was also utilized for the immersion tests.

Our results investigated Mg degradation with different surface roughness under cell culture conditions. Mg degradation has been involved with both thermodynamic and kinetic mechanisms governing a series of electrochemical reactions. In the initial stage, the Mg immersed in the DMEM with 10% FBS (pH value: 7.3) led to the anodic and cathodic reactions as described by Equations (4)–(6) [56]. Our results indicated that a slight increase in pH value could be observed in the medium (Figure 3a). With the degradation occurring, the interaction between the Mg surface and the electrolyte-containing aqueous media caused the degradation layer formation. Moreover, SEM-EDS indicated the presence of several elements such as Mg, C, O, Na, P, Cl, and Ca (Figure 2a). This can be confirmed that these elements were detected in the degradation layer, which was derived from chemical reactions with the components of DMEM with 10% FBS. Herein, the degradation layer might be composed of MgO, Mg(OH)_2_, MgCO_3_, and serum-related organic components, as previously reported [55].

Anodic reaction: Mg → 2Mg^2+^ + 2e^−^ (4)

Cathodic reaction: 2H_2_O + 2e^−^→ 2OH^−^ + H_2_↑ (5)

Overall reaction: Mg + 2H_2_O →2Mg^2+^ + 2OH^−^ + H_2_↑ (6)

Generally, the in vitro degradation behavior was determined by the applied body fluids. In this experiment, the artificial saliva was neutral to a slightly acidic environment (pH value: 5.5). The main differences between DMEM and artificial saliva were the degradation products and the overall rates. According to the SEM images, the relatively dense and thick degradation layers on the Mg surfaces were formed in the artificial saliva (Figure 2b). The EDS results indicated that the predominant components were Ca, P, O, and C elements. According to the main composition of artificial saliva (Table 1) and the primary degradation mechanism (Equations (4)–(6)), the released Mg ions could further react with calcium ions and phosphate ions, depositing a mixture of a calcium-phosphate layer (i.e., Ca_3_(PO_4_)_2_, Ca_8_H_2_(PO_4_)_6_·5H_2_O and CaHPO_4_·2H_2_O), as previously described [57]. In addition, Mg degradation in the artificial saliva was two-fold lower than its counterpart in DMEM with serum. Compared to DMEM, the original artificial saliva is a slightly acidic solution, which apparently decreases the Mg degradation. This result mainly contributes to the fact that the stable passivation calcium-phosphate layers can be formed in the acidic solution [57,58]. Additionally, based on the degradation mechanism above, the surface roughness had no apparent impact on the degradation products on the samples.

Notably, surface roughness plays diverse roles in the Mg degradation rates and modes under various degradation conditions; however, the overall tendency clearly showed that the degradation rate of the rough Mg specimen (Sa > 2.0 µm) in DMEM was significantly higher than that of the smooth Mg specimen (Sa > 0.2 µm). Firstly, the rough surface exhibited higher values of Sa and Sdr, suggesting that a higher surface area was exposed to the electrolyte. This factor could directly enhance the degradation rate of rough Mg, consistent with a previous finding [32]. Moreover, there was no statistically significant difference in the degradation rate in DMEM despite a pronounced increase in Sa value from P400 to P180. This probably arose from the fact that the surface roughness is critical to predicting degradation rates, which degradation kinetics are also influenced by surface free energy, compactness of passivation layers, and protein absorption. In comparison, the surface roughness did not affect Mg degradation in the artificial saliva (Figure 3b). This could be attributed to the stable passivated calcium-phosphate layer formed on the Mg surfaces, preventing chloride ion attacks on the matrix [56]. In addition, regarding the degradation modes, uniform degradation was observed among the Mg specimens with different surface roughness, irrespective of the applied simulated physiological media. Our finding is inconsistent with previous studies, which reported that a rough Mg surface leads to the severity of pitting corrosion in the initial degradation stage [59,60]. This discrepancy could be attributed to the high purity magnesium with grain refinement, which escapes the micro-galvanic corrosion affected by the alloys [61].

### 4.2. Cytocompatibility Affected by Surface Roughness

In this study, the cytocompatibility of high-purity Mg was investigated through a combination of an extract test and a direct contact test. The results of the extract test indicated that cell viability was not influenced by Mg with different surface roughness. Furthermore, the direct contact test revealed that fewer viable cells were attached on the smooth Mg surface than those on moderately rough and rough Mg surfaces (Sa > 1.0 μm).

Basically, the standardized extract test is a projection of the material’s degradation products towards cellular responses, mainly involving the released degradation products and related cellular tolerances [62]. The quantitative results depicted that all sample extracts had no adverse effects on three different cell lines, indicating no cytotoxic effects according to the requirements of ISO standard (Figure 4). This indicated that cells could become tolerant of the toxicity effects of degradation products. According to the analysis of sample extracts (Table 2), the increased Mg ions and hydroxide ions were observed, which can be considered as the main degradation products from the sample extracts. Previous studies reported no apparent decrease in cell viability when the pH value of the medium was less than 9 [63,64]. In addition, the Mg ion concentrations (<285 μg/mL) in the tested sample extracts were lower than the overall cellular tolerance (<360 μg/mL), as previously reported [40,65]. It is noteworthy that the sample extracts with surface roughness had no effects on the cell viability, attributed that there were no apparent differences in the degradation products released from Mg having diverse roughness surfaces.

The direct contact test investigated the cellular behavior on the material interface, which is mainly determined by material degradation, surface characteristics, and cellular response. In our preliminary experiments, the cells were nonviable on bare (or untreated) surfaces, mostly consistent with most previous studies [66,67]; therefore, high concentration and rapid release of Mg ion should be responsible for the cytotoxic effects. Undoubtedly, most cells (i.e., osteoblast, macrophage) have no chance to directly grow on the original surface of Mg-based implants (without any protein adsorption and the formation of an initial degradation layer) under most physiological conditions [68]. Herein, specimens were pre-incubated before the tests to mimic the initial degradation layer formation, as previously described [67,69].

Regarding Mg-based alloys, surface roughness is regarded as a critical parameter to predict cellular response against the interfaces of Mg-based implants. Our results showed that the rough Mg surfaces exhibited cellular attachment superior to the smooth ones. As to the bioinert materials (i.e., Ti and PEEK), it is well-known that a moderately rough surface (Sa > 1.0 μm) could improve cell adhesion, proliferation, and differentiation because of optimized surface topography and surface-free energy [70,71]. In addition, a recent study demonstrated that the rough surface with the ridge/valley network feature of Mg-based metals could efficiently facilitate the proliferation of endothelial cells while suppressing the smooth muscle cells [24]. In addition, a more stable passivation layer formed by rough roughness could be another factor, decreasing the adverse effects of the rapid ion release [72]; therefore, the rough surface could improve cell adhesion with the synergy of the above factors.

Regarding the potential applications, the biodegradability of high-purity magnesium can safely degrade without producing obvious toxic products, making it one of the most promising candidates for biodegradable craniomaxillofacial implants. Surface roughness modification can adjust their degradation kinetics. In the present study, our results demonstrated that the degradation rate of high-purity magnesium with Sa value of surface roughness ranging from 1.0–2.0 µm was appropriate and clinically acceptable. Considering the implant application of high-purity Mg in vivo, a moderately rough surface (Sa between 1.0–2.0 µm) can be used to improve cell adhesion and proliferation; therefore, the results of this study provide significant information for the surface modification of high-purity magnesium implants to enhance cell adhesion and optimize biological responses, especially for the oral and maxillofacial area.

## 5. Conclusions

In the present study, the effect of surface roughens on the in vitro degradation behavior and cytocompatibility of high-purity Mg was investigated. Within the limitations of the current in vitro study, the principal findings were drawn as follows:

1. With increasing surface roughness, the degradation rate of the Mg specimen in the DMEM with FBS was significantly increased. However, no marked increase was observed in the degradation rate when Mg was immersed in the artificial saliva.
2. The degradation mode and the products of high-purity Mg were not obviously affected by the surface roughness.
3. The extract test revealed that Mg extracts derived from different surface roughness did not exhibit any cytotoxic effect on the L929 fibroblast, the MC3T3-E1 preosteoblast, and the RAW264 macrophage.
4. The direct contact test demonstrated that the surface roughness of high-purity Mg with the Sa value > 1.0 μm had the potential to improve cell attachment.

In summary, the present study indicated that the high-purity Mg with a micro-roughness range of Sa between 1.0 and 2.0 μm had an optimized balance between biodegradability and cytocompatibility, especially for the craniomaxillofacial applications. Nevertheless, the limitation of our present study is that the semi-static immersion test is not able to fully simulate the dynamic conditions of fluid circulation. In addition, although the work investigated the interaction between surface roughness and cell adhesion, further insights into the correlation between surface topography/chemistry and biological signals are further required.

## Figures and Tables

**Table 2:** Analysis result of sample extracts.

Sample	Mg Ion Concentration (μg/mL) *	pH Value
Control	19.2 ^1^	7.56 ± 0.06
P180	242.8 ± 29.5	8.02 ± 0.03
P400	284.8 ± 26.8	8.09 ± 0.06
P4000	262.8 ± 69.1	8.10 ± 0.04

* Mg ion concentration (μg/mL) determined. ^1^ Data are given from Ref in [53].

## Data Availability

The data presented in this study are available on request from the corresponding author.
